# Psychological distress in pregnancy and postpartum: a cross-sectional study of Babol pregnancy mental health registry

**DOI:** 10.1186/s12884-023-06024-3

**Published:** 2023-11-14

**Authors:** Shahnaz Barat, Azita Ghanbarpour, Seyyedeh Mahboubeh Mirtabar, Farzan Kheirkhah, Zahra Basirat, Hoda Shirafkan, Angela Hamidia, Faezeh Khorshidian, Davood Hosseini Talari, Zeynab Pahlavan, Sedigheh Esmaelzadeh, Zinatosadat Buzari, Mahtab Zeynalzadeh, Shahla Yazdani Charati, Fatemeh Shafizade, Mahsima Adnani, Fatemeh Amirkhanloo, Maedeh Mollaalipour, Atieh Chale kani, Mania Amiri, Razieh Khazaei, Seyedeh Shabnam Mehdinia, Fatemeh Basirat, Simin Asadollahi, Asieh Khodami, Alireza Azizi, Fatemeh Nasiri-Amiri, Nooshin Fatery, Shirin Shahrokhi, Banafshe Zarinkamar, Sajedeh Aligoltabar, Mahbobeh Faramarzi

**Affiliations:** 1 Department of Obstetrics and Gynecology, Infertility and Reproductive Health Research Center, Health Research Institute, Babol University of Medical Sciences, Babol, Iran; 2 Department of Obstetrics and Gynecology, Health Research Institute, Rouhani Hospital, Babol University of Medical Sciences, Babol, Iran; 3 Research Clinical Psychology, Student Research Committee, Health Research Institute, Babol University of Medical Sciences, Babol, Iran; 4 Department of Psychiatry, Social Determinants of Health Research Center, Health Research Institute, Babol University of Medical Sciences, Babol, Iran; 5 Social Determinants of Health Research Center, Health Research Institute, Babol University of Medical Sciences, Babol, Iran; 6 Department of Midwifery, School of Nursing and Midwifery,Infertility and Reproductive Health Research Center, Reproductive Health, Health Research Institute, Babol University of Medical Sciences, Babol, Iran; 7 Midwifery, Clinical Research Development Unit of Rohani Hospital, Health Research, Institute, Babol University of Medical Sciences, Babol, Iran; 8 Clinical Psychology, Student Reasearch Committee, Behshahr Azad University, Behshahr, Iran; 9 Clinical Psychology, Clinical Research Development Unit of Shahid Yahya Nejad, Health Research Institute, Babol University of Medical Sciences, HospitalBabol, Iran; 10 Midwifery, Student Reasearch Committee, Babol University of Medical Sciences, Babol, Iran; 11 Department of General Courses, Social Determinants of Health Research Center, Health Research Institute, Babol University of Medical Sciences, Babol, Iran

**Keywords:** Distress, Psychological, Pregnancy, Postpartum, Risk factors, Anxiety Depression

## Abstract

**Background:**

Psychological distress (PD) is a significant issue during pregnancy and postpartum, adversely affecting both children and mothers. This study aims to determine PD's prevalence and risk factors in a large Iranian population sample during pregnancy and postpartum.

**Methods:**

A cross-sectional study was conducted using data from the Babol Pregnancy Mental Health Registry (located in the north of Iran) between June 2020 and March 2021. A total of 2305 women were included, with 1639 during pregnancy and 666 during postpartum. Psychological distress was assessed using the Brief Symptoms Inventory (BSI-18), and data were analyzed using independent t-tests and multiple logistic regressions.

**Results:**

The prevalence of psychological distress, defined by a cut-off score of BSI ≥ 13, was 19% during pregnancy and 15% during postpartum. Multivariate logistic analysis revealed that high-risk pregnancy was the leading risk factor for psychological distress during the antenatal period (*β* = *1.776, P* < *0.001*), as well as its three subscales: somatization (*β* = *1.355, P* = *0.019*), anxiety symptoms (*β* = *2.249, P* < *0.001*), and depressive symptoms (*β* = *1.381, P* = *0.028*). Additionally, women with a gestational age < 20 weeks had a higher risk of psychological distress (*β* = *1.344, P* = *0.038*) and the somatization subscale (*β* = *1.641, P* < *0.001*). During the postpartum period, women residing in urban areas were at higher risk of psychological distress (*β* = *1.949, P* = *0.012*), as well as two subscales: anxiety symptoms (*β* = *1.998, P* = *0.012*) and depressive symptoms (*β* = *1.949, P* = *0.020*).

**Conclusion:**

The high prevalence of psychological distress emphasizes detecting and treating PD during pregnancy and postpartum, particularly in women with high-risk pregnancies. This study suggests that obstetricians and midwives should implement programs to identify women experiencing psychological distress during early pregnancy through postpartum visits.

## Background

Psychological distress (PD), encompassing symptoms of depression and anxiety, is prevalent during pregnancy and postpartum [1]. Research has shown an increased prevalence of PD in certain countries and the aftermath of natural disasters like earthquakes or responses to social events such as the COVID-19 pandemic [1, 2]. Numerous studies have separately examined the prevalence of anxiety and depressive symptoms during pregnancy and postpartum. Approximately 1 in 5 women experience anxiety during these stages [3]. According to the World Health Organization (WHO), around 10% of pregnant women and 13% of postpartum women face mental health issues, predominantly depression or anxiety [4]. A systematic review has indicated a higher prevalence of specific anxiety disorders, such as panic and obsessive–compulsive disorders, during pregnancy compared to the general population [5]. In Iran, the reported rate of psychological distress is 25% for anxiety and 15% for depression among pregnant women between 6 and 40 weeks of pregnancy [6]. Another study in Iran observed a significant increase in depression levels from weeks 9 to 24 of pregnancy, followed by a decrease in weeks 32–34. Additionally, anxiety levels experienced a significant increase from the beginning of pregnancy until the 24th week but remained unchanged in the second and third trimesters [7]. Furthermore, a study in north Iran found that over half of women with high-risk pregnancies experience at least one mental disorder [8].

Psychological distress is associated with adverse pregnancy outcomes [2, 9]. It typically intensifies during the postpartum period, negatively affecting maternal mental health, maternal and family functioning, and infant-child outcomes [10]. Perinatal anxiety and depression affect women's postnatal well-being and may impact the quality of care for newborn infants and their subsequent growth and development [11, 12]. Women are disproportionately affected by mood and anxiety problems, particularly during pregnancy, and mental health difficulties often worsen or arise during this period [13]. A systematic review identified three categories through which prenatal and postpartum maternal anxiety affects infant development: somatic, developmental, and psychological. These findings underscored the association between prenatal and postpartum anxiety and adverse emotional outcomes in children [14].

There is a wide range of risk factors for PD in pregnancy. In a study, women with chronic anxiety or depressive symptoms were four times more likely to develop psychological distress. Additionally, common predictors of both in pregnancy were low social support, high perceived stress, and a history of mental health issues [15]. According to the literature, pregnant adolescents are more likely than pregnant and postpartum adult women to experience anxiety and depressive symptoms [16]. In another study, nulliparous status, young age, and the nuclear family were found as common pregnancy-specific anxiety risk factors [17]. The researchers have examined the factors related to PD during pregnancy and postpartum, showing that pregnant and postpartum women are at higher risk if unmarried, older, and less educated. Also, low social support, unemployment, financial instability, alcohol/tobacco use, and previous psychological problems are associated with distress in pregnant and postpartum women [18].

Current protocols in Iran have several deficits in improving the mental health of women, such as the lack of a valid screening instrument; inadequate training for providers to enable screening and referral of women; absence of standard diagnostic evaluation and treatment in the referral system; no standard protocol for the diagnosis of depression in the referral system; and no protocol for the treatment of depressed women (psychotherapy, pharmacotherapy, or referral to a psychiatrist for hospitalization). It is necessary to design and implement a protocol with sufficient scientific evidence and compare its results with existing conditions. This study offers the potential for comparison with clinics or providers that did not implement the screening protocol to obtain preliminary data on whether screening reduces mental disorders during the postnatal period or increases the rate of treatment for mental health-related issues. Additionally, there is a lack of evidence for the efficacy of screening and treatment of depression in Iranian pregnant women. To our knowledge, there is no pilot protocol study of the implementation of primary screening and treatment of depression in a large Iranian population in antepartum (early and late pregnancy) and postpartum periods. Our study will provide evidence on screening accuracy, which may help inform the development of guidelines for routine screening for depression in Iranian pregnant women. Thus, this study aims to describe the prevalence and risk factors of psychological distress during pregnancy and postpartum in a sizeable Iranian woman sample.

## Methods

### Study design

This cross-sectional study is part of screening phase of the BPMHR ( Babol Pregnancy Mental Health Registry). This is a hospital-based registry for screening and treatment of common mental disorders of pregnant women in Babol City, northern Iran. This program includes all pregnant women (outpatients or inpatients) of three educational hospitals (Rohani, Yahyanejad, Marzikola) of the Obstetrics and Gynecology Department of Babol University of Medical Sciences. The aims of the BPMHR are the identification of psychological distress (depression and anxiety) in pregnancy and postpartum periods, the evaluation of risk assessment and maternal and fetal outcomes, and timely treatment of mental illnesses. BPMHR includes three phases; *screening of anxiety/depression, diagnosis of the disease,* and *treatment of the mental disorders*. The recruitment of the pregnant women has begun in June 2020. The Ethics Committee of Babol University of Medical Sciences (IR.MUBABOL.HRI.REC1401.184) and the Ministry of Health and Medical Education (Iran) approved this project.

### Study population

This study focuses on screening phase of the BPMHR. The objective of this phase is the recognition of psychological distress (anxiety/depression) in pregnancy and postpartum, as well as evaluating fetal and maternal outcomes. We selected the women who entered in screening phase of the BPMHR (from June 2020 to March 2021) as the raw dataset for our analyses. The inclusion criteria for this study were women > 18 years old who completed the Brief Symptoms Inventory (BSI-18) during pregnancy or postpartum periods and those who proceeded with the pregnancy until the delivery of a viable child (≥ 28 weeks). Exclusion criteria were women who terminated the pregnancy before 28 weeks of gestational age suddenly or therapeutically (e.g., abortion, ectopic pregnancy, hydatidiform mole, stillbirth, and severe fetal abnormality). The data from two questionnaires, including demographics and psychological distress, were examined in this study. Demographic data included women's age, education, place of residence, number of pregnancies, gestational age, pregnancy risk (low/high), and history of mental health problems. High-risk pregnancy was defined as the presence of one or more of the following problems: demographic factors (age < 18, age > 35, low/high body mass index), history of medical diseases (such as cardiovascular, pulmonary, renal, and thyroid diseases), the current status of maternal pregnancy risk (gestational diabetes, hypertension, preterm labor, preterm ruptures of membranes, multiple pregnancies, placenta previa, placenta abruption), high-risk behaviors (substance abuse, alcohol, and smoking), and fetal distress like intrauterine growth restriction [19].

Until 31 March 2021, 2385 participants were included in the screening phase of the registry. We cleaned the BPMHR datasets and removed the missing samples and selected the data based on inclusion/exclusion criteria. We excluded 80 questionnaires from the analysis due to low accuracy in filling them. Finally, 2,305 women (1,639 during pregnancy and 666 during postpartum) entered in the analysis.

### Data collection

Data were collected by trained midwives of three educational hospitals. The midwives had a mini-interview with women to invite them to the study and explain its aim. They collected demographic data and maternal and fetal immediate outcomes. Then, they recorded the data on the registry website (www.register.mubabol.ac.ir). Also, the midwives asked the women to complete the BSI-18 to identify the psychological distress screening. They sent the online questionnaires to eligible women via links using SMS. All participants also completed a written informed consent form during the pregnancy/postpartum period before answering the study questionnaires.

The BSI-18 is a frequently employed test in research to identify prenatal and postpartum anxiety as well as psychological distress. It comprises 18 items, including the GSI (max = 72), somatization, depression, and anxiety subscales. Higher BSI-18 scores indicate more psychiatric symptoms [20]. Its psychometric characteristics have been employed in different countries and languages. The BSI-18 Persian version shows good validity and reliability. Its test–retest reliability has been reported as 0.81 [21]. A total distress score of ≥ 10 for males and ≥ 13 for females, out of a possible 72, indicates the need for psychosocial interventions [22]. If the questionnaire score indicated that the woman was suffering from psychological distress (total score of BSI-18 > 13), the midwives called the woman and suggested having a consultation with a mental health specialist. Also, the midwives contacted the participants by phone and then sent the online surveys during the postpartum period (4 to 6 weeks after birth) to assess the mother's psychological distress and the neonates' well-being.

### Statistical analysis

The statistical analysis was conducted using SPSS software (SPSS 21, SPSS Inc., Chicago, IL, USA). The Kolmogorov–Smirnov test was employed to assess the normal distribution of continuous variables. Descriptive statistics were utilized to examine the frequency distribution, mean, and standard errors of the mean. An independent t-test was applied for binary groups (place of residence, history of medical illness, and high-risk pregnancy) to compare anxiety scores based on specific characteristics. At the same time, analysis of variance was used for more than two categories (gestational age, education, and the number of pregnancies). Additionally, post-hoc analysis was conducted using Tukey's tests. Furthermore, multiple logistic regressions were performed to identify risk factors associated with anxiety symptoms, with BSI-18 > 13 serving as the dependent variable and five factors considered as independent variables (age, gestational age, education, pregnancy risk, and residential status). A significance level of *P* < *0.05* was considered statistically significant.

## Results

Table 1 presents the demographic characteristics of prenatal and postnatal women. Of 2305 women, 1639 (71.1%) were pregnant, while 666 (28.9%) were postpartum. The average age of the total population was 30.5 ± 6.17 years. Additionally, 596 individuals (36.4%) had a high-risk pregnancy. The prevalence of depressive symptoms, defined by a cut-off point of 4 or ≥ 4, was 18.4% for pregnant women and 13.4% for postpartum. Based on a cut-off point of 5 or ≥ 5, the prevalence of somatization symptoms was 27.3% in pregnant women and 21.8% for the postpartum period. Anxiety symptoms, as determined by a cut-off point of 6 or ≥ 6, were present in 14.3% of pregnant women and 13.7% of postpartum women. Furthermore, employing a cut-off score of BSI ≥ 13, the prevalence of psychological distress was 19% (312/1639 individuals) among pregnant women and 15% (100/666 individuals) during the postpartum period.

**Table 1:** Characteristics of the population

Variables	Prenatal (*n* = 1639)	Postnatal (*n* = 666)
Age Mean (SD)	30.45 (6.17)	30.40(6.47)
Education (n, %)		
Primary school	80(5.0)	50(7.8)
High school	1034(64.5)	406(62.9)
University	489(30.5)	189(29.3)
Living place (n, %)		
Rural	686(41.9)	309(46.4)
Urban	953(58.1)	357(53.6)
Gestational age weeks, mean(SD)		
< 20	27.49(10.34)	-
21–27	409(21.6)	-
> 28	187(11.9)	-
	969(61.9)	-
Number of Delivery		
0	681(41.5)	21(3.2)
1	673(41.1)	287(43.1)
≥ 2	285(17.4)	385(53.8)
Risk of pregnancy (n, %)		
Low risk	1043(63.6)	427(64.1)
High-risk	596(36.4)	239(35.9)

Table 2 compares average psychological distress based on the demographic characteristics of pregnant women. One-way analysis of variance (ANOVA) results revealed significant differences in the mean gestational age for the somatization (*P* < *0.001, F* = *8.58*) and psychological distress (*P* = *0.019, F* = *3.96*) subgroups. Additionally, regarding the number of deliveries, the mean was only significant for the depression subgroup (*P* = *0.008*, *F* = *4.79*). Tukey's post-hoc test and pairwise comparisons of the means indicated that the average somatization was significantly higher at a gestational age below 20 weeks compared to 28 weeks (P < 0.001). Furthermore, the average psychological distress was significantly higher at a gestational age of 21–27 weeks compared to 28 weeks (*P* = *0.030*). Similarly, the average depression was higher in individuals with more than two births than those with one birth (*P* = *0.015*). Moreover, t-tests revealed that the mean depression was higher in individuals older than 30 (*P* = *0.014*). Additionally, the mean variable of pregnancy risk in all subgroups of somatization, depression, anxiety, and psychological distress was higher in women with high-risk pregnancies than those with low-risk pregnancies (*P* < *0.001*). However, there was no significant difference in education and place of residence (*P* > *0.05*).

**Table 2:** Comparison of mean psychological distress based on characteristics of pregnant women

Variables	Somatization	Depression	Anxiety	Psychological distress	GSI
	Mean (SD) *P*-value	Mean(SD) *P*-value	Mean(SD) *P*-value	Mean(SD) *P*-value	Mean(SD) *P*-value
Age					
18–30	3.80(3.06)	1.99(3.10)	2.77(3.26)	8.58(7.84)	0.48(0.43)
> 31	0.768	0.014	0.498	0.179	0.174
Education (n, %)					
Primary school	3.23(3.18)	2.63(3.57)	3.23(3.31)	9.09(8.44)	0.51(0.46)
High school	3.71(3.06) 0.468	2.20(3.44) 0.115	2.82(3.42) 0.179	8.73(8.45) 0.377	0.49(0.46) 0.359
University	3.65(3.03)	1.89(2.74)	2.55(3.19)	8.14(7.15)	0.45(0.39)
Living place					
Rural	3.58(3.01)	1.98(3.10)	2.72(3.27)	8.30(7.90)	0.46(0.43)
Urban	3.74(3.09) 0.531	2.22(3.34) 0.079	2.76(3.38) 0.393	8.74(8.15) 0.188	0.49(0.45) 0.179
Gestational age (weeks)					
< 20	4.11(3.14)	2.20(3.16)	2.64(3.37)	8.94(8.06)	0.50(0.44)
21–27	3.91(3.37) < 0.001	2.54(3.27) 0.108	3.33(3.53) 0.053	9.78(8.30) 0.019	0.54(0.46) 0.019
> 28	3.38(2.86)	1.99(3.23)	2.69(3.25)	8.09(7.85)	0.45(0.43)
Number of Delivery					
0	3.59(2.98)	1.84(2.74)	2.70(3.13)	8.15(7.13)	0.45(0.39)
1	3.79(3.14) 0.493	2.26(3.41) 0.008	2.72(3.42) 0.629	8.77(8.63) 0.208	0.49(0.47) 0.192
≥ 2	3.61(3.07)	2.51(3.88)	2.93(3.60)	9.08(8.72)	0.51(0.48)
Risk of pregnancy					
Low risk	3.45(2.72)	1.84(2.82)	2.37(3.03)	7.66(7.10)	0.43(0.39)
High-risk	4.07(3.53) < 0.001	2.61(3.82) < 0.001	3.39(3.71) < 0.001	10.09(9.26) < 0.001	0.56(0.51) < 0.001

Table 3 compares average psychological distress scores in the postpartum period based on demographic characteristics. The results of a one-way analysis of variance (ANOVA) indicated that the average number of deliveries significantly influenced somatization (*P* = *0.043, F* = *3.16*), depression (*P* = *0.011, F* = *4.50*), and overall psychological distress (*P* = *0.032, F* = *3.47*). Further analysis using Tukey's post-hoc test and pairwise comparisons revealed that individuals without any childbirth experience had higher mean scores in depression (*P* = *0.009*) and psychological distress (*P* = *0.035*) than those with only one birth experience. Conversely, the mean score for somatization was higher among individuals with more than two births (*P* = *0.043*). In addition, a t-test demonstrated that individuals over 30 years old had a higher average somatization score (*P* = *0.032*). Moreover, urban residents exhibited higher average scores in somatization (*P* = *0.004*), depression (*P* = *0.011*), and psychological distress (*P* = *0.002*) than rural residents. However, education level and pregnancy risk did not have a significant effect (*P* > *0.05*).

**Table 3:** Mean comparisons of psychological distress based on postnatal women's characteristics

Variables	3.71(3.49) 0.032	Depression	Anxiety	Psychological distress	GSI
		Mean(SD) *P*-value	Mean(SD) P-value	Mean(SD) *P*-value	Mean(SD) *P*-value
Age					
18–30	3.00(2.95)	1.63(3.28)	2.69(3.68)	7.32(8.35)	0.41(0.46)
> 31	3.71(3.49) 0.032	1.73(2.85) 0.424	2.69(3.62) 0.926	8.15(8.65) 0.448	0.45(0.48) 0.441
Education (n, %)					
Primary school	2.71(2.24)	1.45(2.49)	2.92(2.53)	7.08(5.91)	0.39(0.32)
High school	3.17(2.84) 0.119	1.47(2.67) 0.053	2.56(3.44) 0.654	7.21(7.41) 0.157	0.40(0.41) 0.140
University	3.69(3.85)	2.15(3.61)	2.84(3.92)	8.70(9.94)	0.49(0.55)
Living place					
Rural	2.71(2.24)	1.48(2.86)	2.24(3.06)	6.70(7.21)	0.37(0.40)
Urban	3.60(3.48) 0.004	1.95(3.28) 0.175	3.14(3.93) < 0.001	8.69(9.09) 0.002	0.48(0.50) 0.002
Number of Delivery					
0	3.59(3.28)	3.60(5.36)	4.50(4.50)	11.90(11.58)	0.66(0.64)
1	3.19(3.09)	1.49(2.68) 0.011	2.66(3.50) 0.080	7.08(7.61) 0.032	0.40(0.42) 0.033
≥ 2	3.52(3.23) 0.177	1.80(3.18)	2.66(3.56)	8.07(8.51)	0.45(0.47)
Risk of pregnancy					
Low risk	3.19(3.09)	1.82(3.21)	2.66(3.60)	7.68(8.65)	0.43(0.48)
High-risk	3.52(3.23) 0.177	1.58(2.91) 0.230	2.85(3.56) 0.397	7.95(7.79) 0.869	0.44(0.43) 0.875

The results of the multivariate logistic analysis for risk factors of psychological distress during pregnancy are presented in Table 4. Based on the results of the multivariate logistic regression, individuals with a gestational age below 20 weeks had a higher likelihood of somatization risk (*ß* = *1.641, P* < *0.001*) and total psychological distress (*ß* = *1.344, P* = *0.038*) compared to those with a gestational age above 20 weeks. Furthermore, women with high-risk pregnancies had a greater likelihood of risk in all subgroups, including somatization (*ß* = *1.355, P* = *0.019*), depression (*ß* = *1.381, P* = *0.028*), anxiety (*ß* = *2.249, P* < *0.001*), and total psychological distress (*ß* = *1.776, P* < *0.001*).

**Table 4:** Results of multivariable logistic regression for risk factors of psychological distress during pregnancy

Predictors	Coding	Somatization	Depression	Anxiety	Psychological distress
		OR(95%CI) *P*-value	OR(95%CI) *P*-value	OR(95%CI) *P*-value	OR(95%CI) *P*-value
Age	18–30 > 31(ref)	1.006(0.77–1.31) 0.968	0.925(0.68–1.25) 0.613	1.055(0.75–1.47) 0.754	1.018(0.75–1.37) 0.908
Education	Primary High/university	0.548(0.24–1.21) 0.137	1.250(0.59–2.62) 0.555	1.185(0.52–2.66) 0.681	1.162(0.55–2.44) 0.691
Living place	Rural Urban(ref)	0.871(0.67–1.12) 0.284	0.933(0.69–1.24) 0.933	0.943(0.68–1.29) 0.720	0.858(0.64–1.14) 0.294
Gestational age	< 20 > 20(ref)	1.641(1.28–2.10) < 0.001	1.088(0.81–1.45) 0.568	1.181(0.86–1.61) 0.296	1.344(1.01–1.77) 0.038
Number of delivery	0 ≥ 1(ref)	0.949(0.73–1.23) 0.696	0.836(0.61–1.13) 0.247	1.049(0.75–1.46) 0.778	0.886(0.65–1.19) 0.424
Risk of pregnancy	Low risk(ref)				
	High risk	1.355(1.05–1.74) 0.019	1.381(1.03–1.84) 0.028	2.249(1.64–3.07) < 0.001	1.776(1.34–2.35) < 0.001

Table 5 presents the results of the multivariate logistic analysis of the risk factors of psychological distress during the postpartum period. According to the findings, individuals older than 31 exhibited a greater risk than those younger in the somatization subgroup (*β* = *1.626, P* = *0.030*). Likewise, urban residents had a higher likelihood of experiencing risk compared to rural residents in the depression subgroup (*β* = *1.949, P* = *0.020*), anxiety subgroup (*β* = *1.998, P* = *0.012*), and overall psychological distress (*β* = *1.949, P* = *0.012*). Furthermore, women with high-risk pregnancies displayed a higher likelihood of experiencing risk compared to those with low-risk pregnancies in the somatization subgroup (*β* = *1.670, P* = *0.027*).

**Table 5:** Results of multivariable logistic regression for risk factors of psychological distress during the postnatal period

Predictors	Coding	Somatization	Depression	Anxiety	Psychological distress
		OR(95%CI) P-value	OR(95%CI) P-value	OR(95%CI) P-value	OR(95%CI) P-value
Age	18–30(ref)	1.626(1.04–2.52) 0.030	0.846(0.48–1.47) 0.556	1.009(0.59–1.70) 0.974	1.158(0.69–1.92) 0.570
	> 31				
Education	Primary	0.550(0.18–1.68) 0.295	0.241(0.03–1.84) 0.170	0.424(0.09–1.87) 0.258	0.186(0.02–1.41) 0.105
	High/university				
	(ref)				
Living place	Rural(ref)	1.488(0.95–2.32) 0.079	1.949(1.11–3.41) 0.020	1.998(1.16–3.42) 0.012	1.949(1.15–3.28) 0.012
	Urban				
Number of delivery	0	0.000(0.000) 0.999	1.613(0.16–16.25)0.685	1.673(0.16–17.18)0.665	1.546(0.15–15.67)0.712
	≥ 1(ref)				
Risk of pregnancy	Low risk(ref)				
	High risk	1.670(1.06–2.63) 0.027	0.750(0.41–1.35) 0.341	1.691(0.99–2.88) 0.054	1.352(0.80–2.28) 0.260

## Discussion

The present study represents the first report of the Iranian mental health hospital-based registry, focusing on the prevalence and risk factors of psychological distress during the extensive prenatal and postpartum periods. BSI-18 and its three subscales (depression, anxiety, and somatization) were employed to assess psychological distress. The study confirmed the high prevalence of psychological distress during pregnancy and postpartum.

The study revealed a high prevalence of psychological distress based on the cut-off score of BSI ≥ 13, with 19% of pregnant women and 15% of postpartum women exhibiting distress. Furthermore, the prevalence of subscales indicated high levels of anxiety (14.3% during prenatal and 14.3% during postpartum), depression (18.4% during prenatal and 13.4% during postpartum), and somatization (27.3% during prenatal and 21.8% during postpartum). These findings align with those of the Italian National Observatory on Women's Health (ONDA), where approximately 90,000 women in Italy experience depressive and anxiety symptoms during the perinatal period, ranging from 10 to 23% during pregnancy and 10% to 40% during the postpartum phase [23]. The prevalence of psychological distress in our study was higher than that reported by Watanabe et al. [24] and Glasheen et al. [18], who found a distress level of 4.8% in pregnant women and 5.4% in postpartum women.

The high prevalence of anxiety and depression in our population can be attributed to several factors. Unlike most studies, our study included inpatients and outpatients, whereas previous studies focused on outpatients. It has been observed that inpatient pregnant women exhibit more symptoms of depression and anxiety compared to outpatients [20]. Additionally, unlike other studies where most women had low-risk pregnancies, half of our study population had high-risk pregnancies. One of the sampling hospitals, Ayatollah Rouhani, served as a high-risk pregnancy referral center in Babol City. Previous studies have shown that pregnant women with high-risk pregnancies experience high levels of stress due to maternal and fetal monitoring procedures such as fetal health tests and ultrasounds [20, 25].

Our research revealed that women who had not experienced childbirth displayed higher levels of psychological distress than those who had given birth, corroborating the findings of Clout and Brown (2015) [26]. Similar results were observed regarding anxiety, as women who had never given birth exhibited higher rates of pregnancy-related anxiety [27]. Several assumptions have been made to explain these findings. Firstly, the lack of pregnancy experience and unfamiliarity with associated challenges may increase anxiety and depression in women experiencing pregnancy for the first time. If coping strategies against stress have not been utilized, the likelihood of experiencing anxiety symptoms is higher in this group. Therefore, it is recommended that midwives and psychologists provide special attention to primiparous women, offering education on the physiological and psychological changes during pregnancy and postpartum periods, child care, and coping strategies to manage stress, specifically during pregnancy [26–29].

According to the current research, women with gestational age < 20 weeks had a higher risk for psychological distress than those above 20 weeks. This finding aligns with Fadzil et al.'s findings, which demonstrated an association between anxiety disorder and a history of mental illness, marital status, unplanned pregnancy, gestational age below 20 weeks, and depression disorder [30]. This observation is consistent with previous studies that indicated decreased depression and anxiety symptoms from the first trimester to 3 months postpartum [31].

This study aims to elucidate the increased likelihood of experiencing anxiety among women in the early stages of pregnancy compared to those in the later stages, with a relative risk of 1.5." In support of this claim, it can be argued that women encounter the initial phase of pregnancy as a period requiring adaptation and adjustment. We propose several reasons for this result. Firstly, at the beginning of pregnancy, women must adjust, ensure acceptance of the baby and maternal responsibilities, and cope with physical symptoms such as morning sickness, back pain, and other pregnancy-related symptoms that may improve over time. Secondly, anxiety in women may be triggered during the first half of pregnancy due to concerns over fetal abnormalities, the stress associated with an unplanned pregnancy, apprehension regarding miscarriage, and the results of screenings during the first trimester. Additionally, considering that half of the women in this study were experiencing their first pregnancy, the stress of adapting to physiological and psychological changes likely contributed to anxiety symptoms.

The present study confirmed high-risk pregnancy as the primary risk factor for psychological distress in pregnant women. This finding is consistent with an earlier study, which identified high-risk pregnancy and gestational age as significant predictors of maternal anxiety in pregnant women [28]. It also indicated higher stress and uncertainty in high-risk pregnancies exacerbate depression and anxiety [32]. Hospitalization may increase the stress levels associated with a high-risk pregnancy. Therefore, hospitalized women with high-risk pregnancies may face a higher risk of depression and adverse neonatal outcomes [33].

The present research found that women who lived in urban areas were at a higher risk for psychological distress than those who lived in rural areas during the postpartum period. On the contrary, some other studies have indicated that rural women have nearly double the prevalence of prenatal depression and anxiety compared to urban and semi-urban women [34]. Several hypotheses have been proposed to explain this result. First, the specific environmental conditions pregnant women face in developing countries help explain this paradox. In developing countries, there is a significant disparity in living standards and facilities between rural and urban populations, although this disparity is less pronounced in developed countries. Second, social and family support may be more prevalent in rural areas than urban areas, contributing to lower anxiety levels among rural women. There is strong evidence that social support is strongly associated with depression and psychological distress during pregnancy, aligning with social support's protective effect [35]. Thirdly, rural women have limited access to obstetricians and psychologists, resulting in inadequate information and support to cope with postpartum stress and care for themselves and their children. These findings suggest the need for further investigations into the causes of high anxiety in rural women during the postpartum period. Additionally, it is recommended to plan interventions to increase social support and reduce postpartum anxiety in rural areas.

The present work has numerous clinical implications in obstetric clinics and wards. The results recommend that obstetricians, midwives, and nurses should pay attention to screening and treating psychological distress in women during pregnancy and after delivery. Also, the midwives and nurses should consider risk factors for psychological distress during pregnancy and postpartum. In high-risk pregnancies, the risk of psychological distress is not limited to pregnancy but is more likely to increase postpartum. Identifying and treating women with psychological distress during pregnancy and postpartum positively affects pregnancy outcomes and reduces the risk of developing or exacerbating mental problems postpartum. Furthermore, timely identification and treatment of patients can reduce long-term complications of hospitalization, treatment costs, and maternal and neonatal complications after delivery.

The study had several strengths. To our knowledge, it was the first evidence of implementing primary screening and treatment of depression using a valid questionnaire in pregnancy and the postpartum period in a large Iranian population. The results of our study indicate that reforms in Iranian maternal care guidelines are necessary. Our study provided evidence on screening accuracy, which can inform the development of guidelines for routine screening for depression in Iranian pregnant women. Our findings propose that routine screening for common mental disorders such as depression and anxiety during pregnancy and childbirth is necessary.

Additionally, our findings open up new possibilities for improving mental disorders in pregnant and postpartum women in Iran. These findings suggest to the Ministry of Health of Iran that depression and anxiety screening with proper tools should be included in the guidelines for maternity care services in both health centers and hospitals. Timely treatment of pregnant and postpartum women experiencing symptoms of anxiety and depression is critical. This study recommends that the general policy of Iran's health system should include appropriate psychological interventions such as psychotherapy and pharmacotherapy (especially telepsychology/telepsychiatry support) or referral to a psychiatrist or psychologist for all pregnant women with mental disorders.

This research had several limitations. First, it was a hospital-based population survey; thus, the frequency of complicated pregnancy was high, and its results may not be generalizable to the low-risk pregnant population. Second, this was a cross-sectional study, making it difficult to determine the exact cause of events and establish a cause-and-effect relationship. A longitudinal cohort study among the general pregnant and postpartum women population is suggested for future research. Third, another limitation was the evaluation of psychological distress with a self-report scale. The study suggests incorporating a clinical interview for diagnosing psychological distress in future studies. Lastly, part of our study was conducted during the coronavirus pandemic, and the stress related to the pandemic may have increased the mental distress of pregnant or postpartum women.

## Conclusion

The study emphasizes that the prevalence of psychological distress is high, with rates of 19% for pregnant women and 15% for the postpartum period. Furthermore, women with high-risk pregnancies, gestational ages < 20 weeks, and residing in urban areas face an elevated risk of experiencing psychological distress. These findings indicate the need for obstetricians and maternity staff to implement programs to identify women with psychological distress during their visits, starting from early pregnancy through the postpartum period. Obstetricians should also prioritize addressing the psychological distress of women with high-risk pregnancies and managing their medical conditions during and after childbirth. Nonetheless, future extensive cohort studies are required to ascertain additional risk factors associated with psychological distress in pregnant and postpartum women.

## Data Availability

The datasets used and/or analyzed during the current study are available from the corresponding author on reasonable request.

## Abbreviations

PD: Psychological distress
BPMHR: Babol Pregnancy Mental Health Registry
WHO: World Health Organization
COVID-19: Coronavirus disease 2019
HADS: Hospital anxiety and depression scale
